# A rare case of subcutaneous emphysema in a young and healthy patient with parainfluenza virus 3 pneumonia

**DOI:** 10.1002/rcr2.70025

**Published:** 2024-09-16

**Authors:** Yahia Yaseen Akeely, Saleh Alesa, Hassan Gafar Hassan, Sultan Almarzouqi, Mohamad Ziad Alchammat, Omar Elghor, Shabana Begum Patel, Emad Hamdi Shaat

**Affiliations:** ^1^ Emergency Medicine Department Security Forces Hospital Riyadh City Saudi Arabia

**Keywords:** emphysema, pneumomediastinum, pneumonia, subcutaneous emphysema

## Abstract

An 18‐year‐old healthy male complained of a 7‐day history of fever, cough, and sore throat, along with a three‐day history of left facial swelling. The x‐rays revealed subcutaneous emphysema in the chest, neck, face, and mediastinum region (Pneumomediastinum). Furthermore, an area of infiltration was visible, indicating pneumonia. Therefore, we immediately started him on intravenous antibiotics. We then moved the patient to an isolation room, considering pulmonary tuberculosis as one of the differential diagnoses. However, the Acid Fast Bacilli (AFB), Mycobacterium Tuberculosis Bacteria‐Polymerase Chain Reaction (MTB PCR), and sputum for gram stain and culture were all negative. On the other hand, the test for parainfluenza virus 3 was positive. The patient was observed with a daily chest x‐ray to monitor the progress of pneumonia and subcutaneous emphysema. Fortunately, the subcutaneous emphysema was significantly reduced on a daily basis until it was completely resolved before discharge home.

## Graphical abstract

We present a rare case of severe subcutaneous emphysema secondary to parainfluenza virus 3 pneumonia.

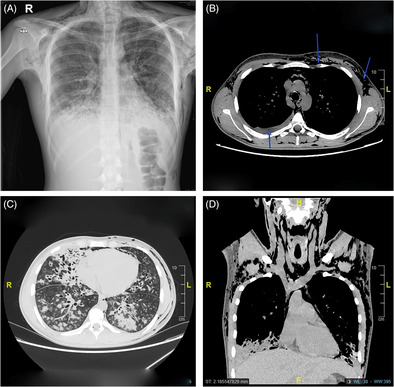

## INTRODUCTION

Subcutaneous emphysema is a rare complication with a variety of etiologies. It is air trapped underneath the skin.^1^ It can be idiopathic or secondary to trauma or non‐trauma causes. Examples of non‐trauma cases include tuberculosis, sarcoidosis, malignancies, bronchial asthma, severe Valsalva, strong coughing, and COVID‐19.^2^ Subcutaneous emphysema is a benign and self‐limited condition, but in rare cases, it may be severe and life‐threatening, mainly when it involves the airway.^3^ The primary treatment focuses on addressing the underlying cause. Surgical intervention can be performed but is rare. We are not aware of any documented case reports for parainfluenza pneumonia complicated with such severe subcutaneous emphysema. Reporting this case aims to illustrate these complications and highlight the treatments and prognosis.

## CASE REPORT

**FIGURE 1:**
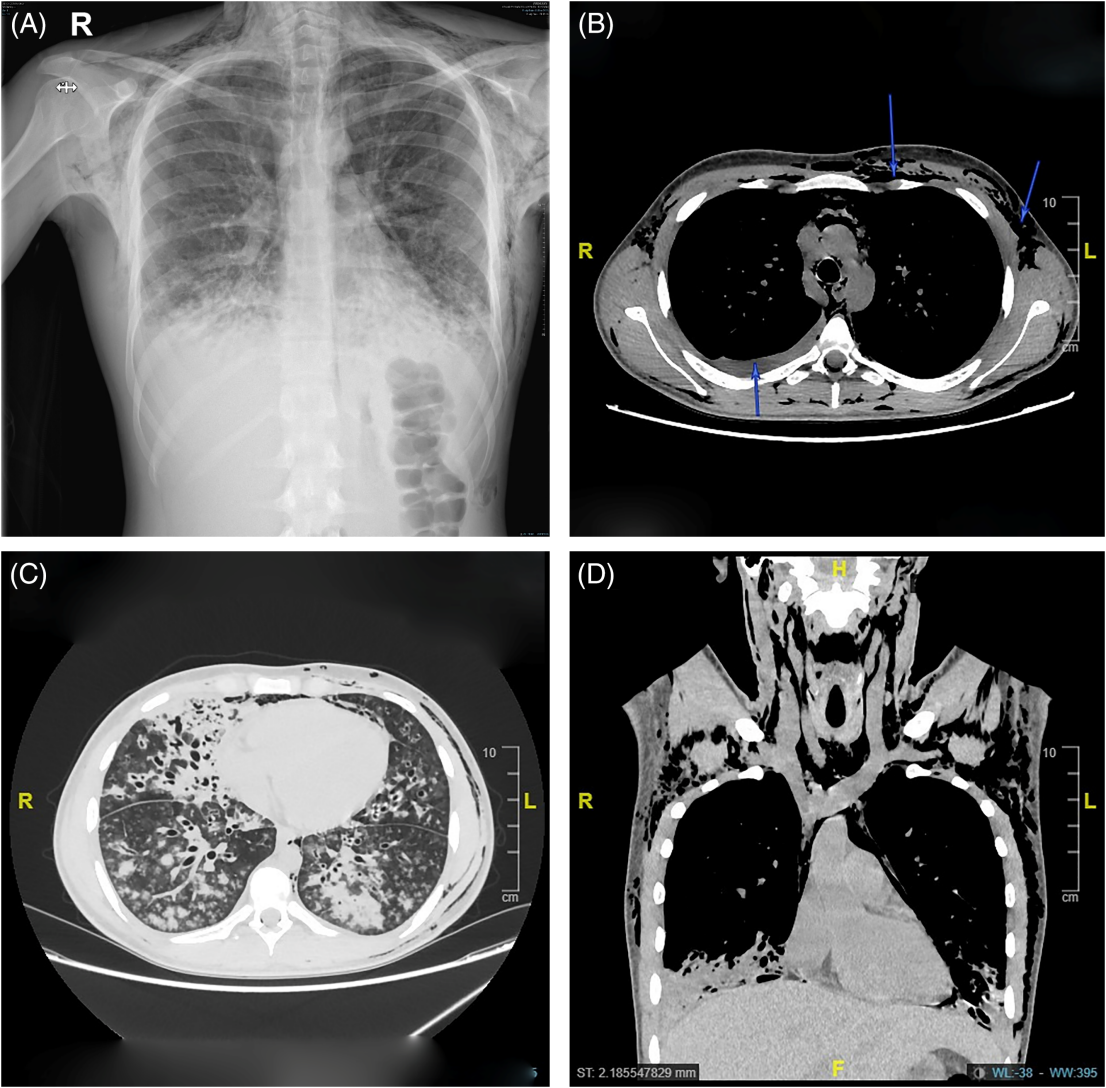
(A–D) presents the images captured on the first day. (A) The chest x‐ray revealed subcutaneous emphysema in the neck and chest. Furthermore, the pneumomediastinum and lung infiltrations bilaterally with obliteration of the costophrenic angles indicate pleural effusions. (B) Computed tomography chest shows subcutaneous emphysema, as indicated by the two arrows above, and pleural effusion, as indicated by the lower arrow. (C) Lung window shows a significant bilateral consolidation, indicating pneumonia and an infectious process. (D) It is a coronal section where we can see the subcutaneous emphysema in the chest, neck, and mediastinum. In addition, there is a consolidation in the lower lobe of the right lung.

An 18‐year‐old male patient presented with a 7‐day history of fever, cough, and sore throat. The primary reason for seeking medical advice was due to facial swelling that had been present for 3 days. On physical examination, he was conscious and oriented, with the following vital signs: blood pressure, 121/80 mmHg; heart rate, 104 bpm; respiratory rate, 19 times/min; oxygen saturation, SpO2 90% on room air; and the temperature, 38.3°C. The emergency physician orders blood tests, x‐rays and start of supplemental oxygen. The chest and neck x‐rays showed significant subcutaneous emphysema with underlying lung infiltrations (Figure 1A). A differential diagnosis of pulmonary tuberculosis (TB) led to the patient's shift to an isolation room. Upon identifying the subcutaneous emphysema on the chest x‐rays, the physician promptly requested a computed tomography (CT) scan of the chest and neck. The CT findings were as follows: there was extensive pneumomediastinum and diffuse subcutaneous emphysema extending along the bilateral chest wall, neck, and upper limbs on both sides, with some air tracking along the spinal canal and the descending aorta to the mid abdomen. The condition was severe because it involved the neck, chest, abdomen, and the arms. There was no pneumothorax. There was extensive bilateral lung bronchiectasis with nodular infiltrates, a tree‐in‐bud pattern, and consolidation. The consolidation encompassed the bilateral lower lung lobes, the middle lobe, and linguar segments, potentially associated with a chest infection (TB). There were also mild pleural and pericardial effusions that did not required any drain. The trachea and main bronchi appeared to be intact. The oesophagus showed no gross abnormality (Figure 1B–D).

We immediately started the patient on intravenous piperacillin/tazobactam and vancomycin. The blood test results were as follows: Leukocytes, 18,100/mm^3^; haemoglobin, 14 g/dL; platelets, 174,000/mm^3^; aspartate aminotransferase, 202 U/L; alanine aminotransferase, 57 U/L; bilirubin, 0.7 mg/dL; prothrombin time, 10 s; partial thromboplastin time, 25 s; C‐reactive protein, 211 mg/L; sputum for AFB‐Acid Fast bacilli; and culture were negative. The polymerase chain reaction for the Mycobacterium tuberculosis bacteria (MTB PCR) was also negative. On the other hand, the test of parainfluenza virus 3 was positive.

**FIGURE 2:**
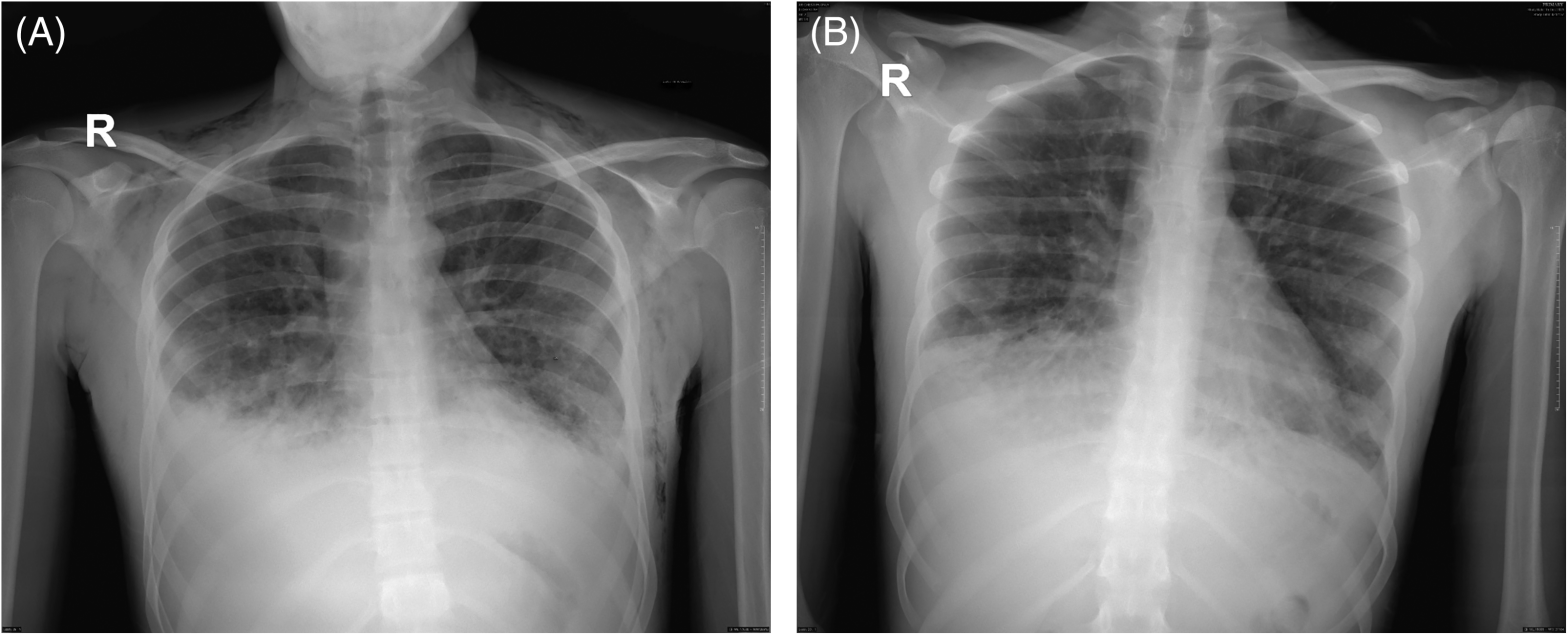
(A, B) shows the x‐ray series over the days of admission. (A) It is the chest x‐ray on day 4 of admission, where it demonstrates regression of the subcutaneous emphysema compared to the 1st day chest x‐ray, as shown in Figure 1A. (B) It is the chest x‐ray on the final day of admission, day 10, where the subcutaneous emphysema resolved completely.

The patient was admitted for intravenous antibiotics and monitored for the disease's progress through daily physical assessments and chest x‐rays (Figure 2A,B). There were no antiviral agents given. There was no surgical intervention to release the subcutaneous emphysema. The treatment was conservative. We discharged the patient with no more subcutaneous emphysema and almost complete resolution of the underlying pneumonia on day 10th of admission. We saw the patient in the clinic 1 month after his discharge, and no image was done on that day of visit. He was doing well with no residual symptoms.

## DISCUSSION

We present a case of severe subcutaneous emphysema secondary to parainfluenza virus 3 pneumonia. The patient was healthy with no previous medical illnesses. The first chest x‐ray indicated subcutaneous emphysema. The most likely cause was the possibility of infectious pathology, given the positive history of respiratory symptoms with fever. Tuberculosis was one of the most likely diagnoses because it is common in our community and also because of its well‐known aggressiveness.Following the workup, the final diagnosis was parainfluenza virus 3 pneumonia. To our knowledge, this is the first reported case of severe subcutaneous emphysema, associated with parainfluenza virus 3 pneumonia. The subcutaneous emphysema was treated conservatively, in addition to appropriate management of the underlying pneumonia. The primary team we consulted included the pulmonary specialist for potential bronchoscopy needs and the thoracic surgeon for potential surgical treatments for the subcutaneous emphysema, if necessary. We also sought advice from gastroenterology regarding oesophageal screening methods to detect any potential leaks. All previous consulted specialties decided not to intervene, but instead to closely monitor the patient and intervene if no improvements were noted.

The cough is another possibility of causing such a complication. Our patient's cough score, according to the Leicester cough score, was 18 where the results range from 3 to 21 where 21 indicates less impairments and severity of cough and 3 indicates the worse and more severe form of cough. In the literature, there are a few case reports that indicate such a possibility.^4^ The other possibility was also COVID‐19, where we can see a Macklin effect. It is a pneumomediastinum caused by alveolar rupture. It is typically seen in trauma or asthma.

Fortunately, the COVID‐19 test result was negative. Because there was no history of such incidences, we excluded trauma and iatrogenic causes. Furthermore, we thoroughly excluded the main possible underlying cause—pulmonary tuberculosis—by conducting all necessary tests.

We present this case because of its extreme rarity, lack of previous publication in the literature, and desire to provide further insight into the conservative treatment option. Most of the time, subcutaneous emphysema is a benign condition.^5^ Treatment of the underlying cause and pathology is of the utmost importance. In a very rare and limited scenario, surgical interventions such as skin incisions, subcutaneous drain catheters, or tunnels may be needed in severe cases that affect the airway, causing ventilatory difficulties or failure.

## AUTHOR CONTRIBUTIONS

The criteria for authorship are met by all authors. The original manuscript was written by Yahia Akeely. The patient's care was the responsibility of all authors, who also contributed to the manuscript's revisions, provided final approval of the published version, and consented to be held accountable for all aspects of the work.

## ETHICS STATEMENT

The manuscript's authors affirm that they have gotten the necessary written consent, informed consent, and permission to publish the accompanying images.

## CONFLIC T OF INTEREST STATEMENT

None declared.

## Data Availability

Data supporting the study's conclusions are available upon request from the corresponding author. Due to privacy or ethical concerns, the data are not made public.
